# High Prescribing: A Case Study of High-Potency Medicinal Cannabis Inducing Psychosis

**DOI:** 10.1155/crps/8870476

**Published:** 2024-12-07

**Authors:** Richard C. J. Bradlow, Sophie Wright, Anamaria Szrajbman Vaz Da Silva, Ferghal Armstrong

**Affiliations:** ^1^Psychiatry, Lismore Base Hospital, Lismore, Australia; ^2^Department of Psychiatry, Monash University, Clayton, Australia; ^3^Turning Point Drug and Alcohol Service, Eastern Health, Box Hill, Victoria, Australia

## Abstract

**Introduction:** There has been a recent significant increase in medical cannabis prescribing in Australia despite weak evidence for its effectiveness in treating the most common indications. Concern has been raised about the potential harms of inappropriate prescription of cannabis; however, there have been no prior published cases of psychosis secondary to medicinal cannabis in Australia.

**Case Presentation:** We present a case of a 21-year-old Indigenous male with psychosis following switching from illicitly obtained cannabis to prescription cannabis, which resulted in Othello delusions towards his partner, violence towards her and ultimately an attempt to end his life.

**Discussion:** Cannabis use is linked to the development of a psychotic illness whether it is prescribed or obtained illicitly. People who are prescribed cannabis are also at an elevated risk of developing cannabis use disorder (CUD). Cannabis prescribers need to screen for risk factors of drug-induced psychosis such as a family member with a psychotic illness, review patients regularly and provide harm minimisation advice to prevent damage from their prescription.

**Conclusion:** There are clear dangers to overprescribing medicinal cannabis and the care that needs to be taken by prescribers to avoid them. There is a need for a change in the regulation of cannabis prescribing in Australia. Further research is warranted on the effects of the increase in prevalence of cannabis prescribing.

## 1. Introduction

Medicinal cannabis became legal in Australia in November 2016 via a special access scheme. Initially, prescriptions were not very prevalent; however, since 2019, there has been a large increase, and now, there are 4000 new special access scheme approvals a month [1]. The reason for this increase is unknown, but market factors rather than clinical need may be a significant driver [2]. The most frequent indications for prescription of medicinal cannabis in Australia are anxiety disorders and insomnia, despite limited evidence supporting its effectiveness for those conditions [1, 3, 4].

Prescribing cannabis for psychiatric disorders is concerning because research indicates that delta-9-tetrahydrocannabinol (THC) can exacerbate positive, negative, general and total symptoms on the Positive and Negative Syndrome Scale (PANSS) [5], even in individuals without psychiatric histories [6]. The Royal Australian and New Zealand College of Psychiatrists (RANZCP) has warned that medicinal cannabis is being overprescribed for disorders with insufficient evidence for its use [7].

The RANZCP has also warned against the risk of psychosis and advised that prescribers screen for risk factors such as a previous history of psychiatric conditions, particularly when prescribing high-potency medicinal cannabis products. Although there have been anecdotal reports in the media about an increased prevalence of psychosis related to the rise in medicinal cannabis prescribing [8], no cases have been published documenting psychosis secondary to medicinal cannabis use.

## 2. Clinical Record

A 21-year-old Indigenous male was admitted to a public psychiatric unit following an overdose of escitalopram in the context of several months of psychosis characterised by low mood, paranoia, auditory hallucinations and delusional jealousy. His psychosis was precipitated by swapping from illicitly bought cannabis to medicinal cannabis. This was on a background of self-diagnosed anxiety and a family history notable for a sister with bipolar affective disorder.

The patient described an 8-year history of daily cannabis use, smoking approximately 1 g a day, mixed with tobacco through a water pipe. Eight months earlier, he changed from illicitly bought cannabis to prescribed cannabis, with the indication of anxiety. He smoked 1 g of high THC content medicinal cannabis a day through a water pipe and completely ceased smoking illicitly obtained cannabis.

Following swapping to the medicinal cannabis, the patient developed paranoia, auditory hallucinations and delusional jealousy focussed on his partner having an affair. This led to a rupture in his relationship with his partner, who was pregnant at the time, and verbal aggression towards her. Collateral history from his partner was in keeping with this timeline.

The patient developed low mood due to these symptoms and sought a prescription of escitalopram from his primary care doctor, who was different from his medicinal cannabis prescriber. One day later, the patient took an overdose with a plan to end his life. His cannabis prescriber was not aware of him developing the psychotic symptoms as follow-up mental state assessments were not being conducted.

Following his overdose, the patient was admitted to an inpatient mental health unit for 12 days, during which his psychotic symptoms completely resolved with abstinence from cannabis and aripiprazole 15 mg each morning and quetiapine immediate-release 50 mg nightly. He was provided with psychoeducation around substance-induced psychosis and was able to gain excellent insight into what occurred. The patient was discharged with a plan to engage with community mental health supports, abstain from cannabis and continue with his antipsychotics for at least 6 months. The patient's cannabis prescriber was informed of his psychotic episode, and it was requested that they cease prescribing him cannabis.

## 3. Discussion

This paper discusses a case of medicinal cannabis causing psychosis, and although the outcome was positive, a tragic result was narrowly avoided. Cannabis use is linked to the development of a psychotic illness in a dose-dependent manner, and cannabis use disorder (CUD) increases the risk of the user developing a psychotic illness by 3.4 times [9]. For people who already have an increased risk of developing a psychotic illness, such as having a family member with a psychotic illness, this risk will be even higher. This case highlights the potential harms of an unregulated system in which cannabis prescribers are not screening for the risk factors of substance-induced psychosis, such as a family member with a psychotic illness, reviewing patients regularly, or providing harm minimisation advice.

Of people prescribed cannabis, 25% are estimated to have CUD. The risk for CUD is higher among people who use medicinal cannabis daily, use large quantities, inhale cannabis rather than eat it, are young, of male gender, and have another substance use disorder [10]. CUD was likely present in the case discussed here with several of those risks being present. Medicinal cannabis may have a role in the management of CUD as a form of harm minimisation in conjunction with other strategies, such as avoiding daily use, using a vaporiser to minimise damage to lungs and frequent reviews to monitor mental state. However, that was not the indication for prescribing in this case, and harm minimisation advice was not given.

The current model of poorly regulated prescribing may already be resulting in significant harms to patients and could erode trust in medical professionals. It appears to be more influenced by commercial interest than patient needs. A change to regulation is needed to reduce further harms.

Although it is impossible to say definitively that the medicinal cannabis use was the cause of the psychosis of the patient described here, the timing is highly suggestive that it was. It was also the view of two psychiatrists, the patient, and his family that the medicinal cannabis had caused the psychosis.

## 4. Conclusion

This is the first case report of a psychotic episode precipitated by cannabis prescribed in Australia. It highlights the potential dangers of medicinal cannabis prescriptions and the care that needs to be taken by prescribers. There is a need for change in the regulation of cannabis prescribing in Australia and for more research on the effects of the increase in prevalence of cannabis prescribing.

## Data Availability

Data sharing is not applicable to this article as no new data were created or analysed in this study.
